# Supplementary material for: Physicochemical and microbiome changes in queso Crema de Chiapas during ripening
Source: PLoS One. 2026 Jan 7;21(1):e0323038. doi: 10.1371/journal.pone.0323038 (PMC12779153; doi:10.1371/journal.pone.0323038)
Supplement: Attachment — Submitted filename: Response_to_Reviewers_auresp_2.docx [file pone.0323038.s002.docx]

Reviewer #1: The issue of sample pooling remains and weakens statistical inference. Pooling biological replicates before sequencing limits assessment of intra-group variability but at the same time is a "common practice" in many published work. The authors acknowledge this, it still affects the depth of beta-diversity analyses and limits robust conclusions.

Also, the identification of fungal species via ITS amplicons (e.g., Candida etchellsii, C. tropicalis) should be interpreted cautiously, and the manuscript does try to do so but verification through culture-based methods or qPCR for critical genera was possible.

The presence of Aeromonas, Enterobacter, and Chryseobacterium is flagged, but quantification (e.g., CFU equivalents or thresholds) is absent. You mention these as “low abundance,” but you do not define what constitutes a risk threshold.

On fungal species identification from ITS amplicons:

We appreciate the reviewer’s caution regarding the taxonomic resolution of ITS-based analyses. Species-level assignments were interpreted conservatively and supported by concurrent qPCR assays targeting Candida etchellsii and C. tropicalis (now explicitly referenced in the revised text, Fig. 8).

qPCR detection (Ct values = 7.22 and 9.84, respectively) confirmed the presence of these species in the corresponding stages (T1 and T3), reinforcing the reliability of the ITS classification.

While full phenotypic validation through culture isolation was beyond the scope of this metagenomic survey, the qPCR results corroborated the taxonomic classification obtained from ITS amplicon sequencing, providing additional confirmation consistent with the sensory and ripening attributes observed in these cheeses.

On low‑abundance genera and absence of quantitative risk thresholds:

We agree that quantitative thresholds enhance risk interpretation. In this study, the potentially pathogenic genera Aeromonas, Enterobacter, and Chryseobacterium were detected at relative abundances < 0.5 % of total reads, which falls below the detection limits commonly associated with microbial spoilage or infection risk in dairy matrices.

Regarding the genera Aeromonas, Enterobacter, and Chryseobacterium, targeted qPCR assays were performed to verify their presence. The amplification curves for Aeromonas and Enterobacter did not cross the fluorescence threshold (Ct > 35), indicating an absence of detectable DNA. Chryseobacterium showed a weak signal (Ct = 3.26 at T2), consistent with a trace or environmental occurrence rather than viable contamination. Together, these findings confirm that the sequences detected by 16S rRNA amplicon analysis likely represent non‑viable or background DNA rather than active populations. Therefore, we maintain that the presence of these genera is not indicative of contamination risk, but rather illustrates the sensitivity of metagenomic surveillance in detecting trace microbial DNA.

Summary clarification added to manuscript

In response to these valuable comments, we have:

Expanded the Materials and Methods section to justify the pooling strategy and reference supporting studies.

Added Ct evidence supporting C. etchellsii and C. tropicalis detection, reinforcing the reliability of ITS identifications.

Quantitatively defined “low abundance” (< 1 % RA) and stated that potentially pathogenic genera were non‑viable under culture conditions.

We thank Reviewer #1 again for these insightful remarks; integrating them has significantly strengthened the methodological transparency and interpretive rigor of this work.
